# Intraperitoneal adipose tissue is strongly related to survival rate in a mouse cecal ligation and puncture model

**DOI:** 10.1038/cti.2016.3

**Published:** 2016-02-12

**Authors:** Shuhei Niiyama, Osamu Takasu, Teruo Sakamoto, Kazuo Ushijima

**Affiliations:** 1 Department of Anesthesiology, Kurume University School of Medicine, Kurume, Fukuoka, Japan; 2 Department of Emergency and Critical Care Medicine, Kurume University School of Medicine, Kurume, Fukuoka, Japan

## Abstract

Cecal ligation and puncture (CLP) models exhibiting polymicrobial sepsis are considered as the gold standard in sepsis research. However, despite meticulous research being conducted in this field, only few treatment drugs are available, indicating that CLP sepsis models do not completely mimic human sepsis models. The greatest flaw in CLP models is abscess formation because the localization of inflammation caused by abscess formation increases the survival rate. Therefore, by resecting intraperitoneal adipose tissue, we developed a mouse CLP model wherein abscess formation was unlikely. Survival rates at 7 days postoperatively were compared using the Kaplan–Meier method for an intraperitoneal adipose tissue resection group (resection group, *n*=34), an intraperitoneal adipose tissue non-resection group (non-resection group, *n*=35) and a sham group (*n*=10). Results indicated that the survival rate was significantly higher in the non-resection group compared with the resection group. Intraperitoneal macroscopic findings in the non-resection group revealed the localization of inflammation caused by abscesses formation covered in adipose tissue. The survival rate for the sham group was 100%. Measurement of interleukin 6 (IL-6) indicated that during the 12 h after the creation of the CLP model, the median level of IL-6 was 1300 (552–3000) pg ml^−1^ in the non-resection group (*n*=19) and 3000 (1224–8595) pg ml^−1^ in the resection group (*n*=19). Meanwhile, for the sham group, IL-6 values were below measurement sensitivity in most cases (9/10 mice). Thus our results suggest that, in CLP models, intraperitoneal adipose tissue has an important role in abscess formation and is strongly related to the survival rate.

The systemic host response because of sepsis leads to severe sepsis or septic shock as it progresses. These conditions are serious medical issues affecting millions of people around the world each year and further resulting in at least one in four deaths. Currently, this number is increasing.^1^ However, no appropriate method for its clinical treatment has been established, and the disease pathology remains unclear.

To elucidate the mechanism underlying systemic inflammatory responses such as sepsis, various experimental animal models that mimic typical pathophysiological changes observed in sepsis patients have been developed. In particular, cecal ligation and puncture (CLP) models are considered as the current gold standard for animal experiments on sepsis.^2, 3^ This is because CLP models are easy to create, generally offer good reproducibility and show disease progression in a manner similar to humans; these models exhibit rupture of the cecum and perforation of the diverticulum.^4^ However, despite detailed research being conducted using experimental animal models, only few treatment drugs have been developed and are available for practical use.^3^

CLP models have been used for >30 years in pathophysiological research on sepsis.^4, 5^ The results of experiments using CLP models are strongly affected by the following factors during the creation of these models:^2, 3, 6^ (i) ratio of cecal ligation; (ii) number of cecal perforations, size of cecal perforations and pressure toward the tip from the site of cecal ligation; (iii) variability between laboratories; (iv) difference in gender, age and pedigree; and (v) problems associated with the irregularity of the host's immune response promoting abscess formation, which prevents the progression of septic shock.

When creating the CLP model, we often observed the aforementioned abscess formation^7^ and questioned the actual efficacy of the candidate treatment drugs. Intraperitoneal abscess formation is considered to be a major limitation pertaining to CLP models.^3^ Abscess formation arises for some unknown reason and prevent the progression of sepsis, leading to doubts as to whether the candidate treatment drugs have been mistakenly judged to be effective. In fact, Buras *et al.*^2^ also indicated that some candidate treatment drugs may promote initial abscess formation, which could be misinterpreted as a successful treatment for septic shock. Therefore, based on intraperitoneal macroscopic findings, in this study we aimed to verify the hypothesis that the amount of adipose tissue is closely related to abscess formation and that this increases the survival rate. Meanwhile, in recent years, studies have shown that adipose tissue is an important supply source of adipokines, which have both anti- and pro-inflammatory effects.^8^ This point should also be taken into consideration.

## Results

### Survival rates

**Figure 1:**
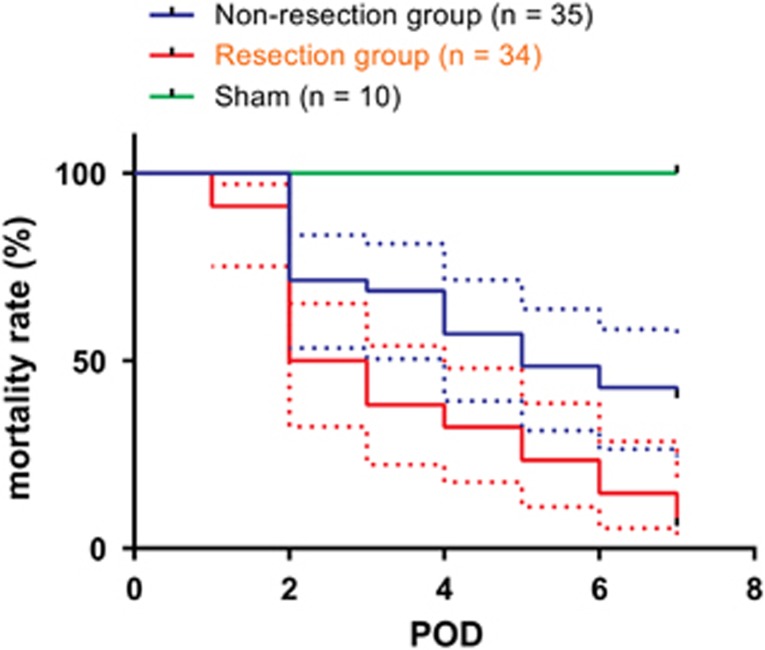
Comparison of survival rates using the Kaplan–Meier method on post-CLP day 7 in the resection, non-resection and sham groups. Data are shown as mean±s.d. The dotted lines show s.d. A significant difference was noted between the non-resection and resection groups (*P*<0.001).

As shown in Figure 1 and Table 1, the results indicated that the survival rate was 40% (14/35 mice) in the non-resection group and 5.9% (2/34 mice) in the resection group. Between these two groups, the survival rate was significantly higher in the non-resection group (*P*<0.001). In the sham group (10 mice), the survival rate was 100%.

### Intraperitoneal macroscopic findings

Adipose tissue in mice is mainly a white, semi-transparent, soft tissue around the testis, large and small intestines and in the kidney area. We focused on adipose tissue in this study because a relatively large amount of this tissue is present around the testis, much of which is found on the abdominal wall side of the large and small intestines.

**Figure 2:**
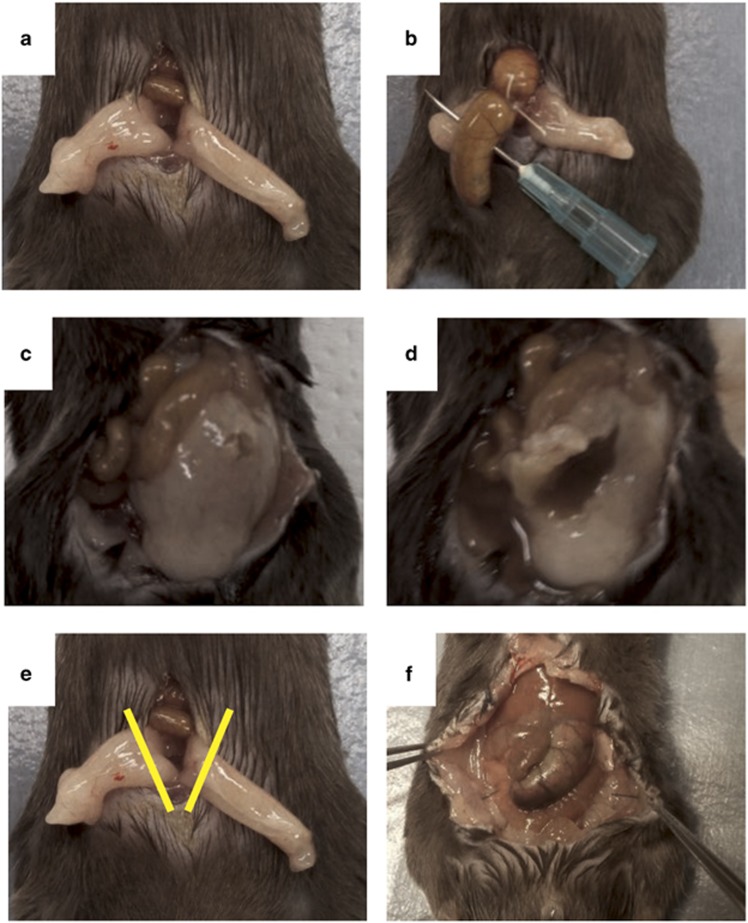
(**a**) Findings showing abdominal adipose tissue exposed outside of the body. (**b**) Ligated using 4-0 silk thread approximately 15 mm from the end of the cecum and perforated using a 21-G needle. (**c**) Non-resection group survival case. Abscess formation findings. (**d**) When the covering in panel (**c**) was resected, pus drainage was noted. (**e**) Yellow lines show the adipose tissue resection in the resection group. (**f**) Laparotomy findings for the resection group (after 12 h).

In the peritoneum of each mouse that survived in the non-resection group, we observed the cecal ligation site to be covered in adipose tissue and an abscess to have formed (see Figure 2c for typical findings). When this covered tissue was removed, a necrotic cecum and an accumulation of pus were noted in all mice (see Figure 2d for typical findings). In 14 of the 21 mice (67%) of the non-resection group that died, the adipose tissue covering of the cecum was insufficient. Although not as clear as in the non-resection group, mice in the resection group that survived also exhibited the gross adhesion of adipose tissue and the surrounding large and small intestines, which caused the intraperitoneal localization of inflammation (data not shown). A necrotized cecum was noted in all mice of the resection group that died but no capsulized abscesses were observed (data not shown).

### Serum interleukin (IL)-6

**Figure 3:**
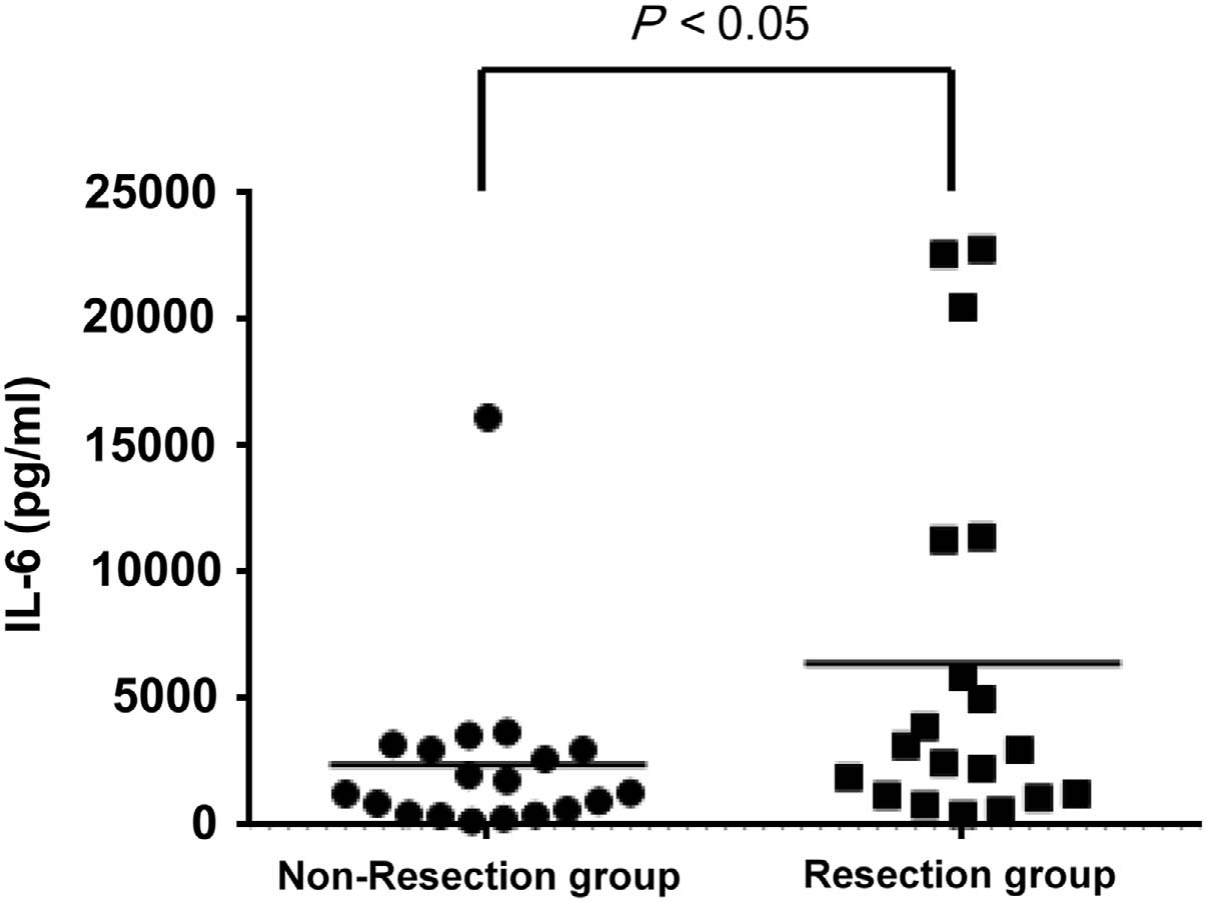
Comparison of IL-6 levels at 12 h after CLP in the resection (*n*=19) and non-resection (*n*=19) groups. Levels were significantly higher in the resection group (*P*<0.05).

Figure 3 shows a scatter diagram for serum IL-6 values in the resection and non-resection groups. The results of one-way analysis of variance indicated that the median value was 3000 (1224–8595) pg ml^−1^ for the resection group (*n*=19) and 1300 (552–3000) pg ml^−1^ for the non-resection group (*n*=19). Thus the median value was significantly higher in the resection group (*P*<0.05).

However, for most mice in the sham group (9/10 mice), IL-6 values were below measurement sensitivity. One of the 10 mice had an IL-6 value of 19.9 pg ml^−1^. Therefore, we were unable to perform statistical analysis.

## Discussion

Sepsis is associated with high onset and fatality rates. Thus appropriate animal models are important for sepsis research.

The findings of this study strongly suggest that intraperitoneal adipose tissue is a significant factor in intraperitoneal abscess formation, which has been considered a major limitation of conventional CLP models. The fact that lack of coverage by adipose tissue was noted at a high frequency in mice that died in the non-resection group is also consistent with our hypothesis. Seven days postoperatively, the survival rate of the resection group was extremely low at 5.9%. This suggested that localization by the remaining abdominal adipose tissue and adipose tissue surrounding the kidneys or adhesion by adjacent large and small intestines was insufficient. These findings suggest that the amount of adipose tissue around the testis very strongly affects post-CLP survival.

IL-6 is a parameter considered to reflect the intensity of CLP inflammation. Results at 12 h after the creation of the CLP model for both groups indicate that IL-6 levels were significantly lower in the non-resection group. Furthermore, postoperatively at 12 h, hyperemia in the large and small intestines wall was more severe in the resection group than in the non-resection group. Here we observed a larger amount of intraperitoneal exudate and greater dilation of the large and small intestines. However, as we did not observe changes over time in IL-6 values in each group, we were unable to consider actual peak values, resulting in this being a limitation of our study.

The characteristics of the model outlined here include the fact that there is a much lower possibility of abscess formation following model creation compared with conventional models, and the model may more accurately mimic panperitonitis symptoms with reproducibility.

However, there are a number of limitations associated with CLP. One of the most important aspects is that results in clinical settings are more significantly affected than those in normal experimental mice owing to multiple factors, including genetic background, treatment intervention, supportive therapy, age and preexisting conditions.^9^

Furthermore, although findings obtained from CLP models can generally be applied to patients who develop sepsis as a result of peritonitis caused by perforation of the abdominal area, it is possible that other pathways are activated by various types of insult, which may progress to sepsis. For example, the frequency of fungal sepsis has increased, which is alarming because it has a poor prognosis.^10^ Other polymicrobial sepsis models include colon ascendens stent peritonitis.^6^ However, even in different types of polymicrobial sepsis models, there are differing model characteristics. Therefore, multiple models must be used to investigate target pathophysiology and enable analytical research from multiple viewpoints. This also appears to be a limitation of sepsis research conducted using a CLP model.

In recent years, studies have suggested that adipose tissue is an important supply source of adipokines, which have anti- and pro-inflammatory effects.^8^ However, as we did not examine adipokines in our model, we consider this to be a limitation and a point that requires further research in the future.

Finally, for animal experiments, the viability, particularly that of conventional models, implies that many animals are required to determine whether the observed effects were really based on this phenomenon.^6^ The high reproducibility (in the sense that abscess formation is unlikely) of our model shows that the number of animals used may be reduced in the future, which could make it an extremely useful model from the viewpoint of ethical considerations in animal experiments. Other notable advantages include the identification and resection of adipose tissue surrounding the abdominal reproductive organs being a very simple surgical procedure accompanied by little bleeding that does not significantly change the operation time (we used an electrical scalpel but ligation is also possible).

## Methods

### Mice

Male C57BL/6 mice (25–30 g; 12–14 weeks) were purchased from Japan SLC, Inc. (Shizuoka, Japan). Mice were kept in a breeding facility with a stable room temperature (23±2 °C) and a 12-h light/dark cycle (light from 0700 am to 1900 hours). Food and water were provided to mice throughout the experiment with no restrictions. Before the experiment, for at least 1 week after purchase, mice were kept in the breeding facility to allow them to regain their strength after being transported. All mice used in this study were handled in accordance with the Guide for the Care and Use of Laboratory Animals, as adopted by the US National Institutes of Health, and the specific protocols were approved by the Institutional Animal Care and Use Committee of Kurume University School of Medicine.

### CLP surgery

The CLP technique was performed in accordance with the methods by Wichterman *et al.*^4^ and Rittirsch *et al.*^11^

First, mice were separated into two groups (resection and non-resection groups). Efforts were made to reduce differences between the groups by dividing mice obtained on the same day into the two aforementioned groups and performing the experiment multiple times (we performed the experiment seven times). We also set up a sham group that underwent only intraperitoneal adipose tissue resection. Weight of the mice was measured to confirm that they were of the specified weight. Subsequently, they were anesthetized using isoflurane (2.5–3%), and this was confirmed by there being no reaction when their hair was removed using tweezers. Mice were placed on their backs and restrained. Anesthetization was maintained using isoflurane (2.5–3%). Next the abdominal areas of mice were extensively shaved using an electric razor. After adequately disinfecting the hair with an Isodine (povidone iodine) cotton swab to prevent infection, the skin of the lower abdomen was pinched using tweezers and a longitudinal skin incision was made while being careful not to perforate the peritoneum with the scalpel. After making the skin incision, surgical scissors were used to expand the opening to the peritoneum by approximately 1.5–2 cm. We identified linea alba (midlines white fascia) of the abdominal musculature and dissected it to create an intermuscular incision and fascial- and peritoneal-layer incision. Here onwards, we outline the method of surgery for the non-resection group. Lower abdominal adipose tissue on both sides was confirmed (Figure 2a), and tweezers were used to remove the adipose tissue (including that covering the male reproductive organs) outside the peritoneum while being careful not to damage any of the organs. Care was continuously taken to prevent adipose tissue from drying using physiological saline solution heated to approximately 37 °C. Subsequently, we quickly moved to create the CLP model. The cecum was extracted using blunt anatomical tweezers or a hygienic cotton swab. During this time, care was taken not to damage any mesenteric vessels. In many cases, the cecum was positioned on the left side of the abdomen. Before the ligation of the cecum, its contents were gently pushed to its tip and to 15 mm from its tip; a 4-0 silk thread was used to perform the ligation (taking care not to ligate the ileocecal valve). Next, a 21-G needle was used to make two punctures (penetrating from the mesenteric to non-mesenteric side; Figure 2b). After removing the needle, a small amount of feces was pushed out from the mesenteric and non-mesenteric side perforations to confirm the patency of the holes. The cecum was returned to the peritoneum while taking care to prevent the spread of feces to areas such as the surrounding skin, and the adipose tissue from both sides exposed outside of the body was returned to cover the middle of the abdomen. Then 4-0 nylon threads were used to perform uninterrupted sutures of the peritoneum, fasciae and abdominal musculature, and the skin was also sutured using a 4-0 nylon thread. Furthermore, 0.05 mg kg^−1^ of body weight of buprenorphine melted in 1 ml of physiological saline solution was subcutaneously injected, and the mice were then returned to the breeding facility. Postoperative painkillers were repeatedly administered every 12 h for 2 days. Next we outline the method of surgery for the resection group. The basic surgical procedure was based on that used for the non-resection group. Points of difference were the fact that, before performing CLP, adipose tissue on both sides of the abdomen was confirmed. Furthermore, the adipose tissue was extracted from the peritoneum using tweezers while being careful not to damage any organs; adipose tissue was resected using an electrical scalpel approximately 5 mm distally from the testes while being careful not to damage the male reproductive organs (Figure 2e).

### Survival rates

The survival rate end points were on the seventh day after model creation (for the resection, non-resection and sham groups). The Kaplan–Meier method was used to analyze survival rates.

### Intraperitoneal macroscopic observation

During this experiment, survival was postoperatively investigated until a maximum of 14 days. Intraperitoneal macroscopic findings were observed in all animals in the non-resection and resection groups. This observation was conducted at the time of death of mice that died postoperatively before 14 days. For mice that survived postoperatively until 14 days, observation was performed at the postoperative fourteenth day through re-laparotomy under anesthesia.

### Cytokine measurement

Elevation of IL-6 is considered to be strongly associated with increased mortality rates owing to sepsis,^12^ and high levels of IL-6 are strongly related to decreased post-CLP survival rates.^13^ Therefore, we measured IL-6 using a commercially available measurement kit (Mouse IL-6 Assay Kit-IBL, Gumma, Japan) and the enzyme-linked immunosorbent assay method. To measure IL-6, we used different mice from those used to observe survival rate to create mouse models that we divided into two groups (resection and non-resection groups). To decrease instability in the experiment, mice obtained on the same day were divided into the two aforementioned groups and the experiment was conducted multiple times (we conducted the experiment four times).

Blood collection was conducted 12 h after CLP creation. Briefly, we performed laparotomy after confirming that a sufficient level of anesthesia had been achieved using isoflurane. The caudal vena cava was exposed and blood was collected using a 26-G needle attached to the tip of a 1-ml syringe. After blood collection, mice were quickly killed by dislocating the cervical region. The collected blood was centrifuged to separate plasma using a BD Microtainer tube (Franklin Lakes, NJ, USA) and stored at −80 °C until measurement. IL-6 was measured twice for each specimen and the mean value was used.

### Statistical analysis

Data are expressed as mean±s.d., median and interquartile range (Q1–Q3) for variables that did not follow a normal distribution or frequencies, as noted. Significant differences according to the Kaplan–Meier method underwent log-rank testing, with *P*<0.05 considered to be statistically significant. Statistically significant differences between the two groups were confirmed using the Mann–Whitney *U*-test (two-tailed test); *P*<0.05 was considered to be statistically significant. The statistical software used was GraphPad Prism version 6.0 for Windows (GraphPad software, San Diego, CA, USA).

## Figures and Tables

**Table 1:** Comparison of survival rates (POD7) in the non-resection and resection groups, proportion of macroscopic intraperitoneal adipose tissue coverage and IL-6 values (12 h after CLP)

	*Non-resection group*	*Resection group*	P*-value*
Survival rate (*n*=69)	40% (14/35)	5.9% (2/34)	<0.001
			
*Proportion of macroscopic intraperitoneal adipose tissue coverage (n=69)*			
Survival group	100% (14/14)	100% (2/2)*	NS
Death group	33.3% (7/21)	0% (0/32)	<0.001
			
Median IL-6 value (pg ml^−1^) (*n*=38)	1300 (552–3000, *n*=19)	3000 (1224–8595, *n*=19)	<0.05

Abbreviations: CLP, cecal ligation and puncture; IL, interleukin; NS, not significant; POD, postoperative day. The proportion of macroscopic intraperitoneal adipose tissue coverage was confirmed when 7 days passed postoperatively in the survival group and by autopsy once death was confirmed in the death group. With regards to asterisk (*), although the proportion of macroscopic adipose tissue was less than in the non-resection group, mice in which remaining adipose tissue or adhesion with the surrounding large and small intestines were noted were considered to be positive for adipose tissue coverage.

## References

[bib1] Dellinger RP, Levy MM, Rhodes A, Annane D, Gerlach H, Opal SM et al. Surviving Sepsis Campaign: international guidelines for management of severe sepsis and septic shock, 2012. Intensive Care Med 2013; 39: 165–228. 23361625 10.1007/s00134-012-2769-8 PMC7095153

[bib2] Buras JA, Holzmann B, Sitkovsky M. Animal models of sepsis: setting the stage. Nat Rev Drug Discov 2005; 4: 854–865. 16224456 10.1038/nrd1854

[bib3] Dejager L, Pinheiro I, Dejonckheere E, Libert C. Cecal ligation and puncture: the gold standard model for polymicrobial sepsis? Trends Microbiol 2011; 19: 198–208. 21296575 10.1016/j.tim.2011.01.001

[bib4] Wichterman KA, Baue AE, Chaudry IH. Sepsis and septic shock—a review of laboratory models and proposal. J Surg Res 1980; 29: 189–201. 6997619 10.1016/0022-4804(80)90037-2

[bib5] Hubbard WJ, Choudhry M, Schwacha MG, Kerby JD, Rue LW 3rd, Bland KI et al. Cecal ligation and puncture. Shock 2005; 24: 52–57. 16374373 10.1097/01.shk.0000191414.94461.7e

[bib6] Schabbauer G. Polymicrobial sepsis models: CLP versus CASP. Drug Discov Today Dis Models 2012; 9: e17–e21.

[bib7] Maier S, Traeger T, Entleutner M, Westerholt A, Kleist B, Hüser N et al. Cecal ligation and puncture versus colon ascendens stent peritonitis: two distinct animal models for polymicrobial sepsis. Shock 2004; 21: 505–511. 15167678 10.1097/01.shk.0000126906.52367.dd

[bib8] Ouchi N, Parker JL, Lugus JJ, Walsh K. Adipokines in inflammation and metabolic disease. Nat Rev Immunol 2011; 11: 85–97. 21252989 10.1038/nri2921 PMC3518031

[bib9] Poli-de-Figueiredo LF, Garrido AG, Nakagawa N, Sannomiya P. Experimental models of sepsis and their clinical relevance. Shock 2008; 30 (Suppl 1): 53–59. 18704008 10.1097/SHK.0b013e318181a343

[bib10] van del Poll T, Opal SM. Host-pathogen interactions in sepsis. Lancet Infect Dis 2008; 8: 32–43. 18063412 10.1016/S1473-3099(07)70265-7

[bib11] Rittirsch D, Huber-Lang MS, Flierl MA, Ward PA. Immunodesign of experimental sepsis by cecal ligation and puncture. Nat Protoc 2009; 4: 31–36. 19131954 10.1038/nprot.2008.214 PMC2754226

[bib12] Vyas D, Javadi P, Dipasco PJ, Buchman TG, Hotchkiss RS, Coopersmith CM. Early antibiotic administration but not antibody therapy directed against IL-6 improves survival in septic mice predicted to die based upon high IL-6 levels. Am J Physiol Regul Integr Comp Physiol 2005; 289: R1048–R1053. 15947070 10.1152/ajpregu.00312.2005 PMC1237117

[bib13] Song M, Kellum JA. Interleukin-6. Crit Care Med 2005; 33: S463–S465. 16340422 10.1097/01.ccm.0000186784.62662.a1

